# Structural effects of the information revolution on tax-funded European health systems and some potential policy responses

**DOI:** 10.1186/s13584-018-0284-2

**Published:** 2019-01-09

**Authors:** Richard B. Saltman

**Affiliations:** Department of Health Policy and Management, Rollins School of Public Health, Emory University, 1518 Clifton Rd NE, Atlanta, GA 30338 USA

**Keywords:** European health systems, Tax-funded health systems, European health policy, health services reform, health systems management

## Abstract

The ongoing information revolution has re-configured the policymaking arena for tax-funded health systems in Europe. A combination of constrained public revenues with rapid technological and clinical change has created a particularly demanding set of operational challenges. Tax-funded health systems face three ongoing struggles: 1) finding badly needed new public revenues despite inadequate GDP growth 2) channeling additional funds into new high-quality provider capacity 3) re-configuring the stasis-tied organizational structure and operations of existing public providers. This commentary reviews key elements of this new information-revolution-driven context, followed by a consideration of seven specific policy challenges that it creates and/or worsens for tax-funded European systems going forward.

## The present-day policy context

Industrial revolutions have consequences. First steam, then electricity, disrupted entire structures of industry and ways of social and political life. Historians have described at great length the economic and political re-ordering that accompanied the first industrial revolution. Friedrich Engels famously wrote his analysis of the English working class in the 1840s while in view out the window of his father’s factories’ belching black smoke [1], while Marx and Engels in 1846 in *The German Ideology* argued that economic transformation was the “base” that drove demands for political and social change [2]. In the United States, Mathew Josephson painstakingly detailed the great social re-ordering created by the arrival of corporatized industry in 1880s America [3]. More recently, the American political scientist Morris Fiorina has written about the dramatic political consequences that followed upon this 1880s economic re-ordering in the US [4].

When William Schockley and his research team at Bell Laboratories perfected the first point-contact transistor in 1947, they set in motion what has become the Third Industrial Revolution, in which computer-based information processing – like steam and electricity before it – has fundamentally re-structured nearly all aspects of 21st Century society. Once again, a fundamental shift in economic production is forcing a thoroughgoing re-structuring of both the social and political landscape.

Framed by this shifting historical context, it is not difficult to see the same process underway in the development of contemporary health policy. Looking backward to, say, 1980, health care systems in 2018 clearly operate in a fundamentally transformed medical, technological, economic, and – in train – health policy and financing landscape generated by this third industrial revolution.

The complexity involved in formulating effective policy to govern health systems has been long acknowledged [5]. A wide range of social policy fields including economic theory, organizational behavior, public management and regulatory policy jockey for prominence with specialist expertise in clinical management, medical science, and epidemiology, all influenced by the concerns of sectoral interest groups such as pharmaceutical companies, staff unions and patient organizations.

Even when technological and financial conditions were structurally stable in the mid-twentieth century, this multi-dimensional policymaking landscape was inherently difficult to negotiate [6] Not surprisingly, politicians tended to move carefully and policymaking typically was incremental in approach, with step-wise outcomes linked to “windows of opportunity” and electoral cycles [7–9] . This intricate governance process has become even more complicated in 2018 as national economies continue to confront fundamental structural change, and health systems find themselves dealing with a wide range of disruptive and transitional quandaries.

This commentary argues that core health policy dilemmas faced by tax-funded European countries directly reflect the globalized impact of the ongoing computer-based information revolution, which has further undercut the necessary domestic economic growth needed to generate additional tax revenues, necessary in turn to fund the new international standard of clinical/medical/service benefits that the computer revolution itself has established. While this information revolution, which started to have an impact on health care systems in the early 1980s, is now in its third stage (sometimes referred to as Internet 3.0) involving machine learning and cloud computing, its impact on tax-funded health systems continues to intensify. As rapidly as publicly funded health systems construct politically acceptable responses to these new pressures, the pressures develop further, re-creating major gaps between what is technically feasible and what public systems can provide, reproducing what continue to be core funding and delivery dilemmas.

It is important to acknowledge that tax-funded health systems and their patients especially in Northern Europe have benefited in varying degree and within political constraints by adopting elements of these new information-based forces, including on a range of technology-enhanced provider services such as diagnostics and e-health and as well as on internal financial management technologies. Moreover nearly all systems have a range of “innovation centers” and “innovative pilot projects” underway and/or under development. Yet in many cases progress on these “innovations” has been slow, and often remains incomplete. Changes tend to be small technical adjustments accompanied by a minor new funding allocation. Rather than being structurally and/or industrially disruptive as has occurred in numerous other more privately driven industries, information-based reforms in tax-funded health systems have been largely absorbed into the previous infrastructure, and have not notably shifted existing institutional configurations or organizational operating methodologies. This absence of more thorough-going impact raises important questions for policymaking as the information revolution continues.

This commentary briefly reviews the core health policy consequences of the current industrial revolution, and then outlines seven specific policy challenges that these core consequences create for tax-funded European health systems.

A key caveat is that the policy challenges identified here are derived directly from the changed economic world that the information revolution has created., Precisely because tax-funded health systems guarantee universal access and coverage, responses to the current policy challenges require measures adequate to maintain those guarantees. Those who themselves prefer different health policy responses by these health systems to those raised in the second part of this commentary will need to demonstrate that their preferred strategies will better resolve what remain central structural challenges created by this changed economic landscape.

A second caveat concerns the relevance of the information revolution for social health insurance (SHI) funded countries, both in Europe and beyond. While some countries (Israel in 1995 and Germany in 2009) have grafted a central national fund onto their social insurance structure, the predominant percentage of overall funding in most European SHI systems remains privately paid, tied to salaries from employees and employers [10–12, 31]. Moreover, service providers in most SHI systems are less likely to be directly managed by politicians in national or regional governments. As a result, the funding and management consequences of the information revolution tend to be differently configured and distributed in social insurance based systems. How these information revolution pressures have affected provider services in SHI systems consequently does not directly bear on an analysis of how effectively European tax-funded systems have been able to respond to these same pressures. Moreover, while such a comparison might provide valuable insights, it would not change the character or structure of the particular policymaking concerns that tax-funded systems currently confront. A similar observation can be made about the impact of information-revolution-based pressures in the mixed public-private funded system in the United States. For reasons both of relevance as well as of length, therefore, the following commentary focuses on current information-revolution-derived policy challenges exclusively within tax-funded European health systems.

Finally, and importantly, this commentary focuses on pragmatic nuts-and-bolts reforms within existing health care systems. As such, it does not address theoretical questions such as the appropriate balance between prevention and cure, between downstream as against upstream disease causality, or the role of social as against allopathic determinants of health. Rather, the purpose here is to raise up for broader debate existing organizational dilemmas that tax-funded European systems will need to more effectively resolve over the next period of years.

## Three disruptive health policy consequences

The continuing rapid advance of computer-based information technology has brought in its wake three tightly inter-connected policy dilemmas for tax-funded European health systems:

1. *Funding/Political*: How to identify and appropriate additional revenues to pay for current levels of clinical and particularly elderly-focused health and social care, as overall economic growth continues below that of previous recoveries and tax revenues remain static;
2. *Clinical/Medical*: How to upgrade existing medical services with continually advancing information-based or computer-generated technologies to meet the rapidly rising international standard of care (small and large medical capital equipment; computerized procedure suites such as catheterization and robotic surgery; immunotherapy, biosimilar and now CAR-T pharmaceuticals; gene-based personal medicine);
3. *Organizational/Managerial*: How to transform static/fixed public sector provider institutions into dynamic innovative models of provider behavior and patient service (integrating social care with health services; developing new intermediate-level service, care, and health professional arrangements; adopting novel treatments and procedures; reducing structural bottlenecks inside secondary and tertiary providers).

These three core challenges permeate service delivery across these systems even as more traditionalist academics both defend what they view as the necessary centrality of publicly funded, owned and operated health systems in providing essential health care services to their national citizenry, while simultaneously disparaging the value of past public system reforms including innovative managerial tools such as New Public Management [13, 14] and alternative capital funding mechanisms such as the Private Finance Initiative in England [15].

The current set of operational policy challenges across tax-funded European health systems is particularly demanding. Health system structures and managerial strategies that were revamped in the 1980s and 1990s to introduce stronger steering and greater patient choice of provider have fallen increasingly out of phase with a rapidly changing external policy environment. Lack of long-term economic growth combined with stasis-oriented provider organization has created a growing gap between the staffing and clinical capabilities of existing institutions in increasingly sharp contrast to internationally defined diagnostic and therapeutic standards.

Among the consequences in, as one example, the English NHS, have been extensive delays in adopting new medical technologies and procedures [16], as well as – once again [17]– increasing numbers of cancelled operations and lengthening waiting times for both physician visits and medical treatment [18, 19]. In Sweden, 22% of patients in 2010 had to wait 4 months or more for elective surgery despite the 2005 introduction of a promised 3 month maximum wait [20], and – a more current example - the national government had to intervene in March 2017 with a special 500 million Swedish crown fund (about $65 million US dollars) to support county-level funding for maternity services [21], due to an increased infant death rate and some pregnant women being sent to Finland to deliver [22].

Hoping to soften this structural misfit, policymakers in a number of tax-funded health systems have introduced a series of meso-level administrative reforms and/or reform proposals, starting from before the 2008 fiscal crisis. These include initial hospital re-centralization in Norway in 2002 [23] followed by further recentralization proposals in 2016 [24], with a similar pattern of governmental tightening of controls in Denmark starting in 2007 [25] . There also are ongoing recentralization efforts in Finland [26]; continued discussions in Sweden [27–29]; decentralization pilots in England [30]; and, conversely, the failure of efforts to replace tax-based funding with national health insurance in Ireland [31] .

In Italy, the national government took operating control in 2008 of 6 of the 19 Italian NHS regions due to poor clinical and financial performance [32]. As of January 2018, cancer patients continue to migrate across the country in search of a regional administration that still has funds for drugs they need, and middle-class women sometimes need to sell their houses to pay privately for life-extending breast cancer drugs [33].

As this technologically driven revolution has broadened its impact across society, deeper levels of organizational change inside health systems have become increasingly necessary. These structural challenges threaten to upset longstanding accommodations between powerful health sector logics and actors, however they need to be addressed even in the most change-adverse health systems and by the most traditionalist stakeholders. This pressure for institutional reform continued even as stronger European economies at long last experienced in 2017 some measure of economic recovery from what has been for most EU member states ten lost years since the onset of the 2008 financial crisis [34].

Thus tax-funded European health systems face three tightly inter-connected struggles: 1) to raise additional public funds for their health and social services; 2) to channel additional funds efficiently into new technology and facilities at the provider level; and 3) to reform existing inertial provider organizations inside the public sector. While some of these new funds will replace resources that would have been available earlier had the 2008 crisis not occurred, they will likely continue to be insufficient given the scale of the service delivery challenge that countries confront.

Continued low rates of productivity growth in most European economies suggests that the most recent level of economic improvement may not be sustainable [35]. The English Chancellor forecast in March 2018 that economic growth will remain below trend for the next 4 years [36] and other countries with tax-funded health systems like Finland and Italy also continue to have longstanding economic growth difficulties. Even Norway has stated that the drawn-down rate from its massive sovereign wealth fund, which in budget year 2018 will provide a full 18% of national expenditure (which includes all health spending), is unsustainable [37] . If the 2018 economic slowdown in Eurozone economic growth becomes extended [38–40], pressures for structural reform in tax-based health systems will intensify further.

## Seven practical policy challenges

This section reviews seven practical policy challenges that directly derive from the core policy consequences just detailed of the current information-driven revolution. All seven challenges reflect present-day health system pressures, and thus involve re-thinking day-to-day operations going forward inside most tax-funded European health systems. For each policy dilemma, the section describes the nature of the challenge, and the particular issues it creates for present health sector actors. While these dilemmas are interconnected, they each necessarily require separate policymaking attention.

### #1. Finding a more sustainable balance between ethics and finance

Despite the 2017 economic upturn in Europe, health sector decision-makers in tax-funded health systems in Northern and Southern Europe remain under strong pressure to find better ways to address the large and increasing gap between patient expectations of what is clinically possible, as compared to the inadequate amount of available public health sector funding.

Evidence of this fiscal misfit is widespread [41, 42] . As noted earlier, there continue to be long patient queues for initial physician visits, for specialist visits, for diagnostic testing (particularly high technology studies such as CT and MRI scans), and for elective procedures (particularly cancer operations). Costly new pharmaceuticals are rationed out to only some clinically appropriate patients.

In Sweden, some cancer patients now move their families across county lines in order to get earlier access to life-saving drugs [43], and Summer 2017 figures on prostate operations show that, nationally, only 5 to 10% of procedures are performed within 60 days after diagnosis [44].

NHS England cut off funding in 2015 for 16 new cancer drugs, some 40% of its total, lacking adequate finance for its Cancer Drug Fund, which had been established in 2010 [45]. A 2018 report from the Office of Health Economics found that up to one million NHS patients each year were required to undergo the additional risk of major surgery due to inadequate availability of less invasive keyhole-based equipment and staff [46]). In January 2018, NHS England cancelled all (over 50,000) elective procedures across the entire NHS system for the entire month of January, citing lack of beds and operating room capacity in the “winter flu season” [18, 47] . As of August 2017 (before the January 2018 cancellations), there were over 4,000,000 patients on NHS waiting lists [48, 49]. The British Medical Association has warned that scheduling a primary care visit with one’s listed general practitioner (GP) may require up to 21 days, as 1 in 8 GP posts remain unfilled [50]. Anecdotal evidence indicates that the wait can be up to 30 days in some Southeastern England locations [51, 52].

The Republic of Ireland combined its four health boards into one, and was planning to jettison tax-based funding by moving to a social health insurance based financing system [31] . However a change of government has led to a renewed tax-based structure [53, 54].

Finland’s national government, pressured by recent recession in addition to the problems created by the 2008 financial crisis, is preparing to consolidate public health administration in 2020 from 320 municipalities and 20 hospital districts into 18 elected counties, within which starting in 2020 all health and social services will be combined into one set of planning and paying hands [26, 55].

As a useful comparator, in the Netherlands (which has a social insurance based system), King Willem-Alexander announced to Parliament in September 2013 that “our labor market and public services are no longer suited to the demands of the times,” and that “the classical welfare state is evolving into the ‘participatory society’” in which citizens will be expected to take care of themselves or to develop civil-society solutions for problems like retiree welfare [56]. The 2006 Dutch health insurance reform placed greater financial responsibility directly on the individual, enabling the individual to reduce premium costs by forming informal purchasing groups and through lower utilization rates [57]. New legislation implemented in January 2015 mandated that Dutch municipalities take formal responsibility for elderly home care and nursing home services, with new, strict controls over admissions to nursing facilities expected to increase the responsibility of individuals and families to take care of their elderly members [57].

Although service rationing and re-structuring efforts in tax-funded health systems can help balance health system budgets in the short term, they are likely inadequate over the longer term. This resource gap leads to difficult questions of how new funding can be raised, and from whom. In England, for example, multiple proposals were raised again in Fall 2017 to increase income taxes in order to provide additional funding to the poorly performing English NHS [58–60]. A study by the Institute for Fiscal Studies released in May 2018 concluded that maintaining and improving existing service levels in the NHS would require a tax increase of a additional 10% of all earned income over the next 15 years [61]. In November 2018, the UK’s Conservative government pledged to raise the English NHS's budget by 20.5 billion pounds over the next 5 years, however even that amount has been deemed insufficient [62], and the new revenue will likely require taxation tradeoffs including less (not more) funding for already underfunded social care services [63]. Yet raising general taxation further damages the rate of economic growth, suppressing the earnings available to tax especially given widely held concerns about post-Brexit economic performance. Moreover, higher taxes would be levied despite the fact that England already in 2016 spent about 10% of its total GDP on health care, which is at the EU average rate [64].

These differing revenue-raising proposals have widely varying ethical and equity dimensions, extending from an hypothecated tax that all adults pay, to a special national insurance surcharge on the elderly, to taking funds from the sale of elderly peoples’ homes to compensate for long term care services [60, 65]. With rationing or downgrading of service quality, some patients will lose access to prescribed services, requiring in turn some of those who can afford it to go private and pay out of pocket. With new or increased co-payments and deductibles, low and middle income patients would potentially pay more from their limited incomes. With increased public tax levies, taxpayers across all income brackets would pay more regardless of the services they use, and overall economic growth is retarded. How these decreased services/increased costs will be allocated across different social and economic groups, and in particular the impact on low income and elderly and/or otherwise vulnerable populations, will inevitably incur intense political scrutiny [8, 66].

Taken overall, European governments with tax-funded health systems now find themselves forced by rapid information-revolution-driven change in both their national economy and in the structure of clinical health care (reinforced by the aging of their populations) to consider explicitly re-balancing the ethical and funding settlements that have undergirded their public delivery systems. While such a re-allocation of societal responsibility is historically not unusual in either tax-funded or social insurance funded health systems [67], the challenge in this instance will be to act before the next economic slowdown imposes additional service constraints.

### #2. Developing better strategies to steer structural diversity

Tax-funded European health systems typically set their service objectives to be uniform in character. Most systems have legal and/or political obligations to provide the same standard of services to all citizens wherever they live in the country. Moreover, they usually seek to provide those services themselves, through a comprehensive network of publicly built, publicly operated, and publicly staffed provider institutions. The only major exception has been primary care, delivered mostly by private GPs (England, Denmark; Norway since 2002; Sweden 50% private GP visits since 2011). Here too, though, primary care services have often been publicly provided in publicly built and operated health centers (Sweden, Finland, Spain, and Italy, also in some urban areas in Denmark and England).

These public providers – especially public hospitals – have since the 1980s had difficulty keeping up with the rapidly evolving international standard in technology, as well as with growing patient demand for timely services (shorter waiting lists), higher quality care, greater patient safety, and better outcomes [68–70]. These pressures have increased over the last decade under the combined impact of the accelerated information revolution and the associated 2008 fiscal crisis [58, 71].

These difficulties led countries in the 1990s and 2000s to diversify some aspects of their previously uniform approach to health service provision. On the capital development side, measures adopted included the Private Finance Initiative in England, with over 100 hospitals built [72, 73] and a parallel, more recent use of special purpose private companies in Sweden (Karolinska University Hospital in Stockholm, also Angelholm hospital in Skane County and the Queen Silvia Children’s hospital in Goteborg) and in Finland (Helsinki Children’s Hospital).

On the service delivery side, tax-funded systems have, in varying degree, diversified by collaborating with non-governmentally-operated providers, as well as by co-operating with and/or sometimes themselves establishing more locally responsive intermediate-level outpatient care facilities. There has been increased contracting out of elective procedures and also custodial care to private providers. The English NHS, as one example, has contracted out a substantial number of scheduled surgeries since the 1990s [74]. Recently adopted in some tax-funded countries (Denmark, Norway) has been the inclusion of private hospitals into the public reimbursement scheme [75, 76]. In some countries, long term care beds have been provided primarily by private contractors. European Commission statistics from 2011 show Ireland with 65% of all residential care places as for-profit private and another 9% as not-for-profit private. Equivalent figures from England are 76 and 16%, although they are much lower in the Nordic Region: in Sweden 17% private (both for-profit and not-for-profit) and in Norway 4 and 6% [77].

While these public sector initiated changes have helped, they have been insufficient to meet current service delivery challenges. First, public systems have had difficulty in integrating existing private sector providers into ongoing public sector decision-making and management [78–80]. Public sector providers tend to continue to operate as traditional bureaucracies. Rather than integrating private contractors into broader strategies of care provision, these contractors essentially become catch-basins for the public sector’s clinical overflow. As a result, rather than stimulating efficient and higher quality modes of operation in public sector providers, these private contractors become bulwarks against patient criticism, in effect protecting the public sector from needed clinical and organizational change [70].

Second, tax-funded public systems presently require substantially more diversity of provision types and provider agents in order to have adequate medical capacity in areas such as minimally invasive robotic surgical and also three-dimensional imaging technologies, so as to capture for their patients the benefits of the latest technology-driven quality, safety, and outcomes capabilities. There also is growing demand on public delivery systems to co-operate with and/or themselves establish more locally responsive, intermediate-level outpatient care facilities. 2012 reforms in Norway and Denmark setting up municipal observation units for chronically ill elderly [81], as well as the establishment of “narsjukvarden” care centers in a few counties in Sweden, suggest that public sector providers also can provide some of these more flexibly structured services. However, to satisfy rising patient expectations as well as international standards of clinical care, publicly run health systems will likely increasingly need to affiliate with a range of innovative not-for-profit and for-profit providers, in a variety of different ownership, contract and payment relationships, and in different delivery settings including inpatient as well as elderly nursing home and home care.

With continuing constrained public sector finances yet rapidly changing information technologies, European tax-funded health systems will require extensive links with intermediate level non-public providers. Referring again to The Netherlands as a social health insurance system comparator, its not-for-profit private hospitals have in recent years established some 280 “independent treatment centers” for “less complex, routine care,” with prices 15–20% lower than in the hospitals themselves [57, 82].

Further, public patients in tax-funded health systems will increasingly seek the ability to receive care in a variety of different extramural provider settings operated and located more conveniently for the patient: local urgent care centers that see patients on a walk-in basis 7 days a week 12 h a day; vaccinations and other standard shots at the local pharmacy; and outpatient specialist services at a locally located satellite hospital outpatient center open nights and Saturdays (delivery innovations, it may be noted, that exist currently in many parts of the United States).

Diversity will also mean different public as well as private not-for-profit and for-profit provider owners: community groups, foundations, cooperatives, small local entrepreneurs, large international companies, risk capital funds, etc. While publicly operated hospitals will remain at the core of these delivery systems, they will no longer monopolize them.

Lastly, increased operational diversity means responding to changed conditions quickly and flexibly with alternative strategies. In January 2018, the 900-bed tax-funded public hospital in Atlanta, Georgia - Grady Hospital - faced with a large flu-driven influx of patients that threatened to overwhelm its emergency department, responded by renting a specialized trailer fitted out as an emergency room with 14 patient bays separated by curtains, and hiring the necessary new personnel to staff it. The trailer handled 83 patients on its first day, relieving the pressure on ER beds, and over the flu season helped the hospital guarantee appropriate quality of care to its patients [83, 84]. This flexible approach can be contrasted with that of the English NHS, whose public hospitals, overwhelmed by a particularly bad “winter flu season,” responded by cancelling all elective surgery in all public hospitals for the entire month of January [18, 47, 85].

The following two examples of re-designed service delivery arrangements – one not-for-profit, the other for-profit - highlight the potential to creatively re-think one currently inadequate public sector service - elderly care services - in a manner that expands and/or re-combines delivery of normally separated elderly-related services to provide a more attractive, higher quality of care. Both business models also develop additional lines of activity as a way to improve the company’s total revenues and longer-term financial viability.

Innovative provider #1: *Saffier de Residentiegroep* (saffiergroep.nl) in Den Haag, The Netherlands, is a private not-for-profit nursing home group that, at its Den Haag nursing home facility, also provides both hospice care in house as well as outpatient visits to neighborhood elderly, including home care and home primary care visits. The nursing home’s dining room is designed and built to be a public restaurant, has menus with prices, free wi-fi, and a candy counter, particularly aimed to attract nearby college students for lunch as a way both to earn non-care-related income as well as to provide a more lively environment in the home for their elderly residents. The home also has turned its parking lot into a vegetable garden, inviting nearby junior high school students to help elderly patients with the heavy digging in return for learning how to garden from them.

Innovative provider #2: *Natali* (natalihealthcare.com) in Tel Aviv, Israel is a for-profit private company that simultaneously provides extensive home care and social services in combination with high-tech patient monitoring services. These include:

- Housekeeping services - house cleaning, installation and repair services, meal preparation, laundry services, and more.
- Medical services – emergency and ongoing daily medical needs, medication monitoring and administration.
- Smart sensors for daily routine pattern combine with artificial intelligence software.
- Social services – a weekly social visit, group social activities.
- Daily contact, safety and security services

These Dutch and Israeli examples of innovative elderly-related service delivery, however, are difficult to link up with existing public sector arrangements. The set of cross-cutting services that these companies deliver do not fit easily into the existing health care delivery, staffing, reimbursement, regulatory, or personnel union framework in most tax-funded European health care systems.

Moreover, beyond these specific organizational and payment barriers, in some countries the public sector’s ability to contract with innovative private providers may soon be restricted by new political barriers. In England, Momentum, which now controls British Labor Party policy-making, has characterized the contracting out of both service delivery and new construction as a theft of public resources [86]. The Labor leader, Jeremy Corbyn, and his Shadow Chancellor, John McDonnell, responded to the January 2018 bankruptcy of Carillion - a private contracting company which held over 450 service provision contracts with various public agencies – by announcing that, under a future labor government, all existing private sector contracts for health services, including long-running Private Finance Initiative hospital construction-and-lease-back contracts, would be severed and their activities would be taken back into the public sector [87].

In Sweden, a similar anti-private-sector position regarding health services has been carved out by the governing Social Democratic Party, operating in de facto policy coalition with the Swedish Communist Party, Vanster. These two parties adopted a joint policy statement in September 2014 that explicitly proposed banning “New Public Management” as well as all profit-making in welfare state services [88, 89]. A subsequent Parliamentary committee report in 2017 proposed a ceiling of 7% profit on all public contracts, a figure which has since been contested as generating only a 2% return in labor-intensive health care services, and effectively closing down all private services [44].

### #3. Ensuring better coordination between health and social care

Tax-funded European health systems have become increasingly concerned about their need to better integrate services for their growing numbers of chronically ill elderly. The main policy objectives are seemingly straightforward: a) improve the quality of the social and home care services elderly receive, in order to 2) keep them at home healthier and longer, so as to 3) prevent and/or reduce expensive acute visits to hospitals for emergency room or inpatient somatic care. How to design and implement the needed new strategies, however, can quickly become rather complicated:

- who organizes which level of care (municipal, county, regional, or national governments)
- who delivers it (public home care workers, informal private workers eg legal and/or illegal immigrants; unpaid informal caregivers – typically family members)
- which public sector health provider (visiting nurse; primary care physician, outpatient clinic, inpatient hospital) serves as the medical focal point

In an effort to re-design the overall architecture for serving elderly patients, Finland intends in 2020 to combine its social care (currently run by over 300 municipalities) and all health services (currently organized in 20 hospital districts) into a set of 18 uniform publicly operated “SOTE” or county-level administrations. The presumption is that putting both social and health services under the same administrative roof, with a linked budgeting structure, will cure the lack of coordination that currently exists [26].

Similar efforts to combine services under one administrative roof have been piloted in one location in Stockholm County, the “Tio-Hundra Project,” and other tax-funded health provider systems are exploring similar new strategies and techniques.

There appears to be evidence emerging from an ongoing pilot project in Greater Manchester in England that combining social and health units into a single coordinating structure can produce better outcomes while saving considerable sums. According to a January 2018 commentary in The Times of London newspaper [60],

“Delayed discharges have almost halved and A&E visits are stable, with GPs visiting care homes to reduce the number of ambulances called. The partnership has approved a pay rise for care workers to avert a recruitment crisis, but is still running a surplus.”

Since the Manchester experiment consists of 37 different health or social care organizations, each keeping its separate administrative leadership and budgeting arrangements [30], it would appear that the room for improvement in care coordination and fiscal re-deployment across sectoral boundaries in tax-funded health systems is considerable.

How stable this publicly-delivered organizational coordination will be remains to be seen. Moreover, there have been few new public-private arrangements that bring major innovation onboard or that overcome the hard boundaries that typically re-assert themselves inside public sector organizations [70]. A further issue concerns how publicly operated health systems will be required to shift their cross-organization, cross-government, and cross-provider arrangements to accommodate the growing pattern seen in the United States of replacing generalist primary care physicians/general practitioners (GPs) with specialist geriatricians as the main coordinating doctor for the homebound elderly.

### #4. Overcoming organizational stasis

Organizational and structural rigidities in the public sector are well-known [90]. Most tax-funded health systems have myriad examples of un-rationalized hospitals that cannot coordinate effectively with outpatient services, have operational bottlenecks between clinical and diagnostic departments, have patient medical records that don’t link up with primary care records, and have long-term structural nurse scheduling conflicts, among other coordination and management problems. In turn, institutional relationships between public hospitals and other health providers such as primary and home care tend to be bureaucratic and unresponsive to either intra-organization coordination dilemmas or external changes in clinical or social treatment patterns.

The difficulty that public hospitals have in resolving these issues reflects a core structural dilemma which can be termed “organizational stasis” – that is, an organizational environment that consistently resists efforts at internal reform and/or organizational change. In public hospitals, this type of stasis or “fixedness” can be seen as reflecting a set of three in-built structural relationships:

- the normal dysfunction in all large organizations
- health sector/medical professional dysfunction
- political decision-making dysfunction

and three broader contextual dimensions:

- the complexity of high quality medical care
- market failure in hospital care
- generalized and specific anxiety [70].

The challenge that public health systems confront going forward is to identify strategies that can help overcome these traditional strong tendencies toward inertia without undermining overall organizational stability. Among the key questions to be resolved include:

- How to distinguish between a frozen health system and a stable one?
- How to create a health system that is both a stable and well-functioning but also an innovative operating organization?

While most tax-funded health systems have examples of individual institutions that have made progress on a number of innovation issues, these advances typically do not scale up to the entire system and, in many cases, themselves have relatively short half-lives tied to institutional and managerial obstacles [91].

### #5. Integrating labor unions into change strategies

The role of employee unions in European health systems is a sensitive topic. Hospital managers don’t like to discuss it, politicians downplay it even as they cater to it, and health system researchers largely ignore it.

In publicly operated health systems, however, contract decisions by personnel unions typically influence major operating, quality, and financial dimensions of both tax-funded and social health insurance funded systems. Staffing ratios, shift times, myriad work rules, and other operational dimensions of public providers are as important – and have equal if not more overall institutional impact – as salary negotiations. Moreover, in tax-based European systems, there are specialized union bargaining units for nearly all hospital physicians as well for nurses and other medical staff, which in turn have considerable impact on how medical treatment is organized and delivered. In many instances, these unions – including physician unions – are closely linked to left-of-center labor and/or social democratic political parties, potentially transforming hospital managerial decision-making as well as regional and national health policymaking into an ideological as well as a staffing arena.

Health sector unions have often reacted strongly to public revenue shortfalls created by the economic forces that the information revolution has unleashed [42]. The role of public hospitals as a major employer has further intensified the financial efforts of these unions, as low or no salary increases have effectively frozen overall wages paid to union members. Actual redundancies typically require months, sometimes years, to implement, particularly in the Nordic countries as well as Spain and Italy, and as a result tend not to be initiated by management for just that reason. In Finland, for example, medical staff in public hospitals are considered to have politically approved “posts” from which it is difficult to remove them [92]. Simultaneously, the introduction of new automated technology – both clinical and managerial – can be constrained by existing workrules, making re-assignment of existing personnel to new care modalities a slow sometimes difficult process as well. Looming over all potential change remains the threat of debilitating personnel strikes, which can be about work-rules as much or even more than about salary levels (see, for example, the junior doctor strike against the English NHS in April 2016 [93].

Given this structural misfit between their technological, financial, managerial and personnel contexts, the challenge to publicly funded and/or operated health systems is to develop mechanisms that can turn staff unions into more willing collaborators. While relations have improved in some tax-funded systems over the past 20 years, these European health systems still need better mechanisms to build personnel unions into health system re-structuring processes, integrating unions into the innovative, flexible, fiscally sustainable arrangements that public providers now require:

- Contracts that reward active participation in organizational change
- Contracts that pay incentives to more productive employees
- Quicker re-assignment and redundancy procedures
- Profit-sharing payments to teams/unions, also in public sector

While some of these measures have been introduced in some instances, particularly when the survival of a public provider is at risk, more and broader efforts will need to be made.

### #6. Implementing patient centeredness

Current policymaking interest in Europe in patient centeredness masks substantial nervousness within existing provider systems about what the concept means and how it might alter the existing service delivery landscape. Broadly speaking, the concept of patient centeredness emerged out of the still-ongoing 25 year struggle in tax-funded systems to allow patients to choose where and from whom they receive their primary and specialist care services [68].

Proponents of the original concept of patient centeredness understand it to signal a fundamental shift concerning decisions about the *content* of care – eg. the involvement of patients in deciding what clinical treatment that patient will receive – as well as about the care delivery *process* – eg the involvement of patients in the monitoring of, and, in a number of outpatient monitoring and care processes, in providing certain actual treatment activities. In addition, the advent of personalized medicine, with genetic-code-based pharmaceutical and treatment decisions, adds further to this emerging picture of patient-determined, individually-differentiated health care services.

From an institutional perspective, however, both content and delivery aspects of patient centeredness necessarily require a thoroughgoing re-structuring of how existing tax-funded health care systems configure decision-making to deliver health services. More specifically, it involves two difficult changes: a) a reduction in the power and authority of physicians to decide by themselves on treatment modalities, and b) a ceding of significant political control over decision-making authority about how collective health care resources are to be expended to individual patients. For these two reasons, patient centeredness has increasingly been re-defined in less threatening terms as simply “putting the patient in the center” or as homogenized “person-centered” or “people-centered care” [94], stripping away the original intent of conferring effective decision-making power and authority on the individual patient (or their family representative).

Thus a central challenge to tax-funded European health systems continues to be to find positive organizational and institutional arrangements that can incorporate these new patient-tied computer information, decision-making, and monitoring arrangements into existing delivery structures. Both content as well as process based participation is essential not just for elderly patients, but across health care systems’ entire patient mix, and will increasingly become a key indicator of the overall quality of a system’s care services.

### #7. Incentivizing individuals to improve their own care

The degree to which the intentional behavior of an individual citizen is responsible for their health status and illness state has become the subject of considerable controversy. From germ theory (Pasteur) through clean drinking water (Virchow) to tuberculosis resistance [95] to work-induced stress [96], the degree to which individuals contribute by their behavior to subsequent disease states has been debated and disputed. These disagreements have been further stoked in recent decades regarding the impact of individual behavior such as smoking, eating choices (particularly those that produce obesity), and exercise.

A formative tenet of a tax-funded European health systems has been that, to the extent possible, the financial cost for restorative health services should be collectively not individually borne. This system-wide approach has been based on the belief that it was neither correct not appropriate to blame an individual for the health conditions that he/she had that required care.

This collective responsibility framework has come under increasing scrutiny in recent years. As one example, at the author’s not-for-profit private university in the United States, in a pattern also seen at many for-profit businesses and corporations, faculty and staff are subject to both negative and positive financial incentives concerning their personal health-related behavior.

Negative incentives include a requirement that an employee will have deducted from their salary a penalty amount – in 2018 this is $50/month ($600/year) for the employee and a second $50/month for the employee’s spouse/partner (or $1200/year for a couple)– if the employee and/or spouse/partner uses any type of tobacco product. Employees are required to report their tobacco habits to the university each year, signing a statement indicating that false information is cause for immediate firing. These monthly penalties are exactly the same amount for all employees – there is no financial adjustment for lower paid employees.

Another use of negative incentives – common among employee-contracted insurance in the US – is to require a penalty payment for not keeping a physician or clinic appointment. This is to discourage wasting an expensive medical resource – a physician’s time. At the author’s university, this penalty is typically $50. and it also applies for appointments cancelled less than 24 h. Again, there is no adjustment made based on the employee’s income level.

Positive incentives include up to $500. in health insurance subsidies for employees who have readings taken of their body-mass-index, blood pressure, and cholesterol (tests are provided free of charge by the university).

By comparison to this personal responsibility approach, neither the Swedish nor the Norwegian public health care systems provide an annual physical exam (except for pregnant women or, in Norway, “upon request”) [76, 97]. Most adults in these two socially progressive health care systems do not know their current cholesterol level or, if they haven’t had a medical visit for curative care, their blood pressure or blood sugar levels.

In a policy environment where nearly all European health ministries acknowledge that they have continued smoking, obesity, uncontrolled blood pressure, and uncontrolled diabetes problems, and recognizing that these unidentified conditions eventually lead to expensive medical problems, it would appear appropriate to consider developing carefully constructed negative and positive financial incentives that can be applied inside tax-funded as well as social-health-insurance funded European systems. Similarly, it could be valuable in both health gain and overall cost-of-system terms for European health systems to adopt negative or possibly positive incentives tied to compliance with physician medication instructions for long-term degenerative conditions such as glaucoma, congestive heart failure, and other diseases that, if untreated, result in expensive collective health care expenditures. Precedents here include some regional providers in England [98] and also in Sweden [44] that currently require cardiac surgery patients who smoke to quit before they can be scheduled for a procedure.

Lastly, tax-funded European systems could consider mandating small penalty cash payments for missed hospital outpatient appointments. The cost of one English NHS outpatient appointment has been estimated to be 120 pounds sterling [99]. In the Scottish NHS, a study of all outpatient appointments found that 1,648,421 appointments were missed by patients over a 3 year period, and that 90.9% of those missed appointments were by patients who missed more than one appointment over the 3 year period [100]. These figures suggest that applying individual financial penalties for missed but un-cancelled appointments represents a major efficiency opportunity for tax-funded European systems.

## Conclusions

Tax-funded European health systems face difficult decisions triggered in various ways by the ongoing computer-based information revolution. What was initially felt in the early 1990s to be a peripheral managerial issue – adjusting to a new form of information technology - has now become a central determinant of these systems’ future configuration and, potentially, survival. In this core respect, European health care systems are much like other seemingly mature industries that have found they must rapidly adapt to their new technology-defined environment if they are to thrive, or, perhaps, survive. Just as car manufacturers or food and clothing retailers have had to fundamentally revisit their entire operational models, so to for publicly funded health systems.

It is notable to contrast the slow pace of organizational reform in these European health systems with the rapid structural ferment currently underway in the health sector in the United States. Beyond the multiple intermediary care modalities that have grown up – 7-day-a-week urgent care centers; decentralized specialty imaging, diagnostic, and procedure facilities; vaccinations in pharmacies – there are also ongoing structural re-combinations taking place across major health system sectors. One potentially transformative change is the forthcoming merger of a large pharmacy/pharmacy benefits manager chain (CVS) with one of the US’s largest private insurers (Aetna) [101]. This merger seeks to capture the benefits of advanced information and big data systems in order to provide substantially improved quality and consistency of care while simultaneously reducing per-service health care expenditures [102, 103].

How effective such broad organizational change will be in the very differently structured US health system is difficult to predict. However, this commentary is not seeking to present a case for tax-funded health systems to fundamentally alter their public sector driven characteristics. On the contrary: the argument here is that tax-funded European systems, to continue to remain both clinically and politically viable, will need to respond more effectively to the new economic and medical conditions created by this third industrial revolution. As examples above show from the Netherlands and the United States, also from Norway and Denmark regarding their 2012 re-configuration of public-sector long term care [81], the introduction of new private not-for-profit (a category that once existed extensively in pre-welfare-state European health systems) as well as for-profit actors has the potential to stimulate the innovative institutional change that tax-funded health systems require if they are to continue as universal systems. Raising politically charged arguments about the inherent immorality of all private sector providers will do little to ensure that tax-funded systems resolve the pressures they face or to deflect the otherwise likely erosion of broad middle class support [71] .

Conversely, the discussion in this commentary about the structural effects of the information revolution on tax-funded European systems can usefully serve to trigger a range of broader conceptual questions about health care systems generally. Among the questions that follow from the issues discussed in this article (some previously noted earlier in the text) are a set of wider policy dimensions and implications that emerge from the manuscript’s initial analysis. These include:

- How do European tax-funded funded systems differ in their response to the information revolution from European Social Health Insurance funded health systems;
- What are the ethical implications of moving some health system funding from (higher) taxes to other modes of payment;
- What are the ethical implications of positive and/or negative incentives to citizens to maintain their health, and also to patients to keep their previously scheduled clinical appointments;
- How to distinguish between “static” as against “dynamic” health systems;
- Is it politically feasible in European health systems to engage in, as one reviewer wondered, “rapid and big policy changes US-style”.

Each of these concerns, however, is itself a broad policy topic that requires considerable additional factual and analytic material to be dealt with properly, and which, necessarily, would lengthen this commentary well beyond the space limits of a single journal article. Thus, realistically, this manuscript can only flag up these wider issues as important questions to be discussed by health system analysts going forward.

This commentary has raised seven specific health policy challenges that tax-funded European systems confront as the ongoing information revolution continues in the health sector. These seven challenges have been summarized as the following:

1. *Finding a more sustainable balance between ethics and funding*
2. *Developing better strategies to steer provider diversity*
3. *Ensuring better coordination between social and health services*
4. *Overcoming organizational stasis*
5. *Building labor unions into provider innovation*
6. *Implementing patient centeredness*
7. *Incentivizing individuals to improve their own health*

To achieve these policy objectives, these health systems will need to develop a range of carefully calibrated responses to the pressures they confront. The needed responses as suggested in this commentary can be summarized, in turn, as follows:

1. *Raise new revenue while doing the least economic/social harm*
2. *Harness provider diversity*
3. *Care for more elderly outside of nursing homes/acute care*
4. *Reduce “Stasis” while reinforcing “Stability” in hospital organization*
5. *Entice staff unions to support strengthened innovation*
6. *Expand patient centeredness as the core principle of service provision*
7. *Harness individual financial incentives to encourage personal prevention*

How policymakers for tax-funded European health systems respond to these fundamental institutional and organizational challenges will define essential aspects of future health service provision, and very likely play a central role in these health systems’ continued acceptance by both their patients and citizenry.
